# A Correct Method for Articulating Artificial Teeth in the Mouth

**Published:** 1872-02

**Authors:** A. B. Abell

**Affiliations:** Philadelphia.


### ARTICLE IX.

### *A Correct Method for Articulating Artificial Teeth in the Mouth.*

BY A. B. ABELL, JR., D. D. S., OF PHILADELPHIA.

Having occasionally had cases in which I found it necessary to articulate teeth in the mouth, and having consumed much valuable time in guessing what point needded grinding, I was induced to devise some way by which I could tell promptly what point or points prevented the teeth from coming properly together. Having found the following method to work satisfactorily to myself, I give it to the readers of the *Times:*

Warm a piece of wax, and place it on the cutting edges and grinding surfaces of all the artificial teeth. Then place the case in the mouth, and instruct your patient to close the jaw, until the teeth of the upper and lower jaws antagonize, and press the wax against the face of the teeth while they are in this position. Remove the case from the mouth, and should the wax have become misplaced, carefully replace in position on the teeth. Now secure an articulating model, using an articulator having the movement of the natural jaw. The object in using the wax, it will be seen, is to get an impression of the antagonizing teeth, also the position of the teeth in the mouth when brought together. After the plaster has set, part the case and remove the wax. Then color with vermillion, or other coloring material, the cutting edges and grinding surfaces of the teeth that antagonize with those teeth from which you wish to grind. Bring the articulator together, and the marks made by the coloring material will show you at once exactly what point requires grinding off, and having the teeth in position as they are in the mouth, you will be enabled to see when you have brought them together sufficiently.—*Dental Times.*

